# Treatment outcomes of patients with tuberculosis in war affected region of Khyber Pakhtunkhwa, Pakistan

**DOI:** 10.1186/s12879-020-05184-3

**Published:** 2020-07-01

**Authors:** Tauseef Ahmad, Muhammad Ayub Jadoon, Muhammad Khan, Haroon, Muhammad Mumtaz Khan, Akbar Hussain, Taha Hussein Musa, Muhammad Waqar, Eyasu Ejeta, Manoochehr Karami, Kefyalew Addis Alene, Hui Jin

**Affiliations:** 1 Department of Epidemiology and Health Statistics, School of Public Health, Southeast University, Nanjing, 210009 China; 2 Key Laboratory of Environmental Medicine Engineering, Ministry of Education, School of Public Health, Southeast University, Nanjing, 210009 China; 3 Department of Microbiology, Hazara University Mansehra, Dhodial, 21300 Khyber Pakhtunkhwa Islamic Republic of Pakistan; 4 Department of Microbiology, Abbottabad University of Science and Technology Abbottabad, Abbottabad, Khyber Pakhtunkhwa Islamic Republic of Pakistan; 5 Department of Genetics, Laboratory of Human Genetics, Hazara University Mansehra, Dhodial, 21300 Khyber Pakhtunkhwa Islamic Republic of Pakistan; 6 College of Life Sciences, Northwest University, Xian, 710069 China; 7 Department of Public Health, University of Haripur, Haripur, Khyber Pakhtunkhwa Islamic Republic of Pakistan; 8 Department of Medical Microbiology, Dalian Medical University, 9 Western Section Lvshun South Road, Lvshunkou District, Dalian, Liaoning China; 9 Genome Centre for Molecular Based Diagnostics and Research Centre, Cl-25 Block B Al-Sudais Plaza Abdalian Cooperative Society, Lahore, Islamic Republic of Pakistan; 10 Department of Medical Laboratory Sciences, College of Health Sciences, Wollega University, Nekemte, Ethiopia; 11 Modeling of Noncommunicable Diseases Research Center, Department of Epidemiology, School of Public Health, Hamadan University of Medical Sciences, Hamadan, Iran; 12 Faculty of Health Sciences, Curtin University, Perth, Western Australia Australia; 13 Wesfarmers Centre of Vaccines and Infectious Diseases, Telethon Kids Institute, Perth, Western Australia Australia; 14 Research School of Population Health, College of Health and Medicine, The Australian National University, Canberra, Australian Capital Territory Australia

**Keywords:** Tuberculosis, Treatment outcome, Pakistan

## Abstract

**Background:**

Globally, tuberculosis (TB) remains the leading cause of death from a single infectious disease. TB treatment outcome is an important indicator for the effectiveness of a national TB control program. This study aimed to assess treatment outcomes of TB patients and its determinants in Batkhela, Khyber Pakhtunkhwa, Pakistan.

**Methods:**

A retrospective cohort study was designed using all TB patients who were enrolled at District Head Quarter (DHQ) Hospital Batkhela, Pakistan, from January 2011 to December 2014. A binary logistic regression models were used to identify factors associated with successful TB treatment outcomes defined as the sum of cure and completed treatment.

**Results:**

A total of 515 TB patients were registered, of which 237 (46%) were males and 278 (53.98%) females. Of all patients, 234 (45.44%) were cured and 210 (40.77%) completed treatment. The overall treatment success rate was 444 (86.21%). Age 0–20 years (adjusted odds ratio, AOR = 3.47; 95% confidence interval, CI) = 1.54–7.81; *P* = 0.003), smear-positive pulmonary TB (AOR) = 3.58; 95% CI = 1.89–6.78; *P* = < 0.001), treatment category (AOR = 4.71; 95% CI = 1.17–18.97; *P* = 0.029), and year of enrollment 2012 (AOR = 6.26; 95% CI = 2.52–15.59; *P* = < 0.001) were significantly associated with successful treatment outcome.

**Conclusions:**

The overall treatment success rate is satisfactory but still need to be improved to achieve the international targeted treatment outcome. Type of TB, age, treatment category, and year of enrollment were significantly associated with successful treatment outcomes.

## Background

Globally, tuberculosis (TB) remains the leading cause of death from a single infectious agent (*Mycobacterium tuberculosis*), ranking above human immunodeficiency virus (HIV)/acquired immunodeficiency syndrome (AIDS) [1, 2]. In 2018, an estimated 10 million incident TB cases and 1.6 million deaths due to TB were reported globally [1]. The magnitude of the disease varies from country to country with more prevalent in low-income countries [1, 2].

Increasing the rate of successful treatment outcome is one of the strategies for effective control of TB in the community. The End TB Strategy defines targets for 2030; to decrease the incidence rate by 80% (new cases per 100,000 population per year) and 90% reduction in the number of TB deaths compared with levels in 2015 [3]. However, successful treatment outcome has increased in several countries following the implementation of Directly Observed Treatment Short-Course (DOTS) program [4]. TB is still a major health problem in Pakistan, with an estimated 510,000 new TB cases and approximately 15,000 drug resistant TB cases reported every year [5]. Recently, a standardized TB prevention and control program that regularly monitors the incidence of TB and as well as drug susceptibility testing in the population has been launched at Hayatabad Medical Complex Peshawar, Khyber Pakhtunkhwa province [6]. In Khyber Pakhtunkhwa province of Pakistan several studies have been conducted on the prevalence of TB [7–12]. Monitoring treatment outcomes among war affected countries such as Pakistan provides evidence to assess the effectiveness of TB control programs. There are few epidemiological studies conducted in Pakistan on TB treatment outcomes [13–17]. However, data are limited between 2011 and 2014 in Khyber Pakhtunkhwa for the analysis of trends. Moreover, identifying factors associated with poor treatment outcomes of patients with TB in war affected region provides additional information for national and global TB programs to implement targeted interventions aimed at prevention and management. Therefore, the aim of this study was to assess the TB treatment outcomes and its determinants in Batkhela, Khyber Pakhtunkhwa, Pakistan, between 2011 and 2014.

## Methods

### Study design

A retrospective cohort study was conducted among TB patients who enrolled at District Head Quarter (DHQ) Hospital Batkhela, Pakistan, from January 2011 to December 2014.

### Setting

Batkhela is the capital city of Malakand district and it is one of the popular business city in Khyber Pakhtunkhwa province. Malakand district is situated in Khyber Pakhtunkhwa province. The total population of the district is 720,295 (2017 census) [18]. The DHQ Hospital Batkhela, providing health care facilities to the local residence of Batkhela and district Malakand. The area has been providing humanitarian protection and shelter for a large number of refugees from different districts of Malakand division during flood and war. At the hospital, TB treatment is prescribed based on the recommendations of the national TB guidelines, which is based on recommendations from WHO guidelines. All newly diagnosed TB patients receive a standardized regimen of first-line TB drugs that consists of an 2-month intensive phase with a combination of pyrazinamide (Z), isoniazid (INH), ethambutol (E), and rifampicin (R) a 2-month continuation phase with a combination of rifampicin (R) and ethambutol (E). However, certain groups of TB patients (such as MDR-TB patients) cannot receive the first-line regimen, requiring second-line regimens [19, 20].

### Participants

All TB patients who were enrolled at DHQ Hospital between 1st January 2011 and 31st December 2014 were included.

### Data sources

Data were extracted from patients’ TB registration books and medical records by trained data collectors. The registration books contained basic information such as socio-demographic and clinical characteristics of the patients.

### Variables

Clearly define all outcomes, exposures, predictors, potential confounders, and effect modifiers.

### Laboratory procedure

According to the standard protocol the sputum was collected from the suspected patients having symptom of TB in 5 ml sterile bottle, after collection of sputum the bottle were kept in 15 ml sterile bottle to avoid the leakage of the infectious samples. The samples were labeled and further process by the laboratory technician of the hospitals. Smear microscopy with Ziehl-Neelsen staining and fluorescence microscopy are used in the Hospital for both the diagnosis and monitoring of TB [21].

### Standard definition

TB treatment outcomes and clinical cases were defined according to the standard World Health Organization (WHO) definitions (Table 1). In this study, treatment success was defined as a sum of cured and treatment completed; and poor treatment was defined as the sum of treatment failure, death or lost to follow up.

**Table 1:** Standard definition modified from WHO definitions [22]

Treatment outcomes and clinical case	Definitions
Cured	A patient who was initially sputum smear-positive and who finished treatment with a negative bacteriology result at the end of treatment or was sputum smear negative on two occasions at the end of treatment
Treatment completed	A patient who completed treatment but did not meet the criteria for cure or failure; this definition applies to sputum smear-positive and sputum smear-negative patients with pulmonary TB and to patients with EPTB
Treatment failure	A patient who was initially sputum smear-positive and remained bacteriology or sputum smear-positive at month five or later during treatment
Death	A patient who died from any cause during the course of treatment.
Lost to follow-up	A patient who has been on treatment for at least 4 weeks and whose treatment was interrupted for eight or more consecutive weeks
Transfer out	A patient who has been transferred to another recording and reporting unit and whose treatment outcome is unknown
Successful treatment outcome	A patient who was cured or completed treatment
Unsuccessful treatment outcome	A patient who had treatment failure, lost to follow up, or death
Smear-positive pulmonary TB	A patient with at least two sputum specimens which were positive for acid fast bacilli (AFB) by microscopy, or a patient with only one sputum specimen which was positive for AFB by microscopy, and chest radiographic abnormalities consistent with active pulmonary tuberculosis (PTB).
Smear-negative pulmonary tuberculosis	A patient with symptoms suggestive of TB, with at least two sputum specimens which were negative for AFB by microscopy, and with chest radiographic abnormalities consistent with active PTB, or a patient with two sets of at least two sputum specimens taken at least 2 weeks apart, and which were negative for AFB by microscopy and radiographic abnormalities consistent with PTB and lack of clinical response to 1 week of broad spectrum antibiotic therapy.
Extra-pulmonary tuberculosis (EPTB)	This included TB of organs other than the lungs, such as lymph nodes, abdomen, genitourinary tract, skin, joints and bones, the meninges and others.

### Statistical analysis

The collected data were checked for completeness by principal investor. Data were entered, cleared and descriptive analyses were carried out using Statistical Package for Social Sciences (SPSS) version 20. Binary logistic regression model was used to analyze the association between treatment outcome and potential determinate variables at 95% confidence interval. *P*-value of less than 0.05 was considered as statistically significant.

## Results

### Demographic characteristics of the patients

A total of 515 TB patients, registered and treated for TB at DHQ Hospital between January 2011 to December 2014, were included in this study. Of these, 278 (53.98%) were female and 185 (35.92%) were age less than 20 years (Table 2).

**Table 2:** Characteristics of TB patients attending the DOTS services

Characteristics	Type of TB n (%)			
Sex	Smear positive PTB	Smear negative PTB	EPTB	Total n (%)
Male	120 (50.63)	39 (16.45)	78 (32.91)	237 (46.01)
Female	132 (47.47)	43 (15.46)	103 (37.05)	278 (53.98)
Age				
0–20	74 (40.00)	39 (21.01)	72 (38.91)	185 (35.92)
21–40	100 (52.91)	23 (12.16)	66 (34.92)	189 (36.69)
41–60	46 (56.09)	8 (9.15)	28 (34.14)	82 (15.92)
≥ 61	32 (54.23)	12 (20.33)	15 (25.42)	59 (11.45)
TB patient category				
New	234 (47.46)	79 (16.02)	180 (36.51)	493 (95.72)
Relapse	8 (80.03)	2 (20.00)	0 (0.00)	10 (1.94)
Other	10 (83.33)	1 (8.33)	1 (8.33)	12 (2.33)
Treatment category				
Category-I	243 (48.31)	79 (15.70)	181 (36.51)	503 (97.66)
Category-II	9 (75.00)	3 (25.00)	0 (0.00)	12 (2.33)
Treatment year				
2011	50 (43.10)	26 (22.41)	40 (34.48)	116 (22.52)
2012	59 (50.00)	17 (14.40)	42 (35.59)	118 (22.91)
2013	64 (53.33)	19 (15.83)	37 (30.83)	120 (23.3 0)
2014	79 (49.11)	20 (12.42)	62 (38.5)	161 (31.26)
Total	252 (48.93)	82 (15.92)	181 (35.14)	515

Abbreviations: *PTB* Pulmonary TB, *EPTB* Extra-pulmonary TB

### Clinical characteristics of the patients

Of the total patients, 252 (48.93%) were smear positive PTB, 82 (15.92%) were smear negative PTB and 181 (35.15%) were EPTB as shown in Table 2. Majority of the patients 493 (95.72%) were new TB cases, and 503 (97.7%) were treatment category I (CAT-I). The number of cases diagnosed with TB in the hospital was increased from 116 (22.52%) in 2011 to 161 (31.26%) in 2014.

### Treatment outcomes

The overall TB treatment success rate (i.e. cured and treatment completed) in Batkhela was 444/515 (86.21%). Of all patients, 234 (45.44%) were cured, 210 (40.77%) completed treatment, 14 (2.72%) died, 20 (3.88%) lost to follow-up, and 3 (0.58%) were transferred out (Table 3). The treatment success rate was higher among female 243/278 (87.41%) than male 201/237 (84.81%) and increased over time.

**Table 3:** Trends of TB treatment outcome among TB patients attending the DOTS services

Treatment outcome/year	Year of treatment, n (%)				Overall outcome, n (%)
	2011	2012	2013	2014	
Cured	46 (39.65)	57 (48.30)	60 (50)	71 (44.09)	234 (45.44)
Completed	55 (47.42)	55 (46.61)	52 (43.33)	48 (29.81)	210 (40.77)
Total success	101 (87.07)	112 (94.91)	112 (93.33)	119 (73.91)	444 (86.21)
Died	2 (1.72)	1 (0.85)	2 (1.67)	9 (5.59)	14 (2.72)
Defaulted	0 (0.0)	2 (1.69)	3 (2.5)	15 (9.32)	20 (3.88)
Transfer out	0 (0.0)	0 (0.0)	2 (1.67)	1 (0.62)	3 (0.58)
Unrecorded	13 (11.21)	3 (2.54)	1 (0.83)	17 (10.56)	34 (6.60)
Unsuccessful	15 (12.93)	6 (5.08)	8 (6.67)	42 (26.08)	71 (13.78)

### Factors associated with treatment success

Table 4 shows factors associated with successful TB treatment outcomes. Type of TB, age, and year of treatment commencement were significantly associated with successful treatment outcomes. The odds of successful treatment outcomes was higher among patients with age group 0–20 years (AOR = 3.47; 95% CI: 1.54–4.7.81), 21–40 years (AOR = 2.76; 95%CI: 1.26–6.03), and 41–60 years (AOR = 2.82; 95%CI: 1.08–7.35) compared to patients with age group > = 61 years. The study also found that the odds of successful treatment outcomes was three times higher among smear positive pulmonary TB patients than extra pulmonary TB patients (AOR = 3.58; 95% CI: 1.89–6.78). The odds of successful treatment outcomes were higher among patients with treatment CAT-I than patients with treatment category II (CAT-II) (AOR = 4.71, 95%CI: 1.17–18.97).

**Table 4:** Predictor factors for successful treatment outcome among registered TB cases

Character	No. (%) of TB cases	Successful outcome, n (%)	COR (95% CI)	*P*-value	AOR (95%CI)	*P*-value
Sex						
Male	237 (46.01)	201 (84.81)	0.08 (0.48–1.32)	0.39	0.93 (0.53–1.63)	0.81
Female	278 (53.99)	243 (87.41)	1.00		1.00	
Age						
0–20	185 (35.92)	165 (89.19)	3.34 (1.61–6.93)	0.001	3.47 (1.54–7.81)	0.003
21–40	189 (36.69)	165 (87.30)	2.78 (1.37–5.65)	0.005	2.76 (1.26–6.03)	0.011
41–60	82 (15.92)	72 (87.80)	2.91 (1.22–6.95)	0.016	2.82 (1.08–7.35)	0.034
≥ 61	59 (11.45)	42 (71.18)	1.00		1.00	
TB form						
Smear positive PTB	252 (48.93)	232 (92.06)	2.88 (1.605–5.16)	0.000	3.58 (1.89–6.78)	< 0.001
Sear negative PTB	82 (15.92)	67 (81.70)	1.11 (0.57–2.16)	0.762	1.18 (0.56–2.48)	0.650
EPTB	181 (35.15)	145 (80.11)	1.00		1.00	
Patient category						
New	493 (95.72)	426 (86.40)	0.57 (0.07–4.55)	0.603	0.57 (0.07–4.55)	0.603
Relapse	10 (1.94)	7 (70.00)	0.21 (0.02–2.46)	0.215	0.21 (0.02–2.46)	0.215
Other	12 (2.33)	11 (91.67)	1.00		1.00	
Treatment category						
Category I	503 (97.66)	437 (86.87)	0.21 (0.06–0.68)	0.01	4.71 (1.17–18.97)	0.029
Category II	12 (2.33)	7 (58.33)	1.00		1.00	
Year of treatment						
2011	116 (22.52)	101 (87.06)	2.37 (1.24–4.54)	0.009	2.42 (1.22–4.82)	0.012
2012	118 (22.91)	112 (94.91)	6.58 (2.69–16.09)	0.000	6.26 (2.52–15.59)	< 0.001
2013	120 (23.30)	112 (93.33)	4.94 (2.22–10.98)	0.000	4.31 (1.88–9.86)	< 0.001
2014	161 (31.26)	119 (73.91)	1.00		1.00	

Abbreviations: *PTB* Pulmonary tuberculosis, *EPTB* extra-pulmonary TB, *COR* Crude odds ratio, *AOR* Adjusted odds ratio

## Discussion

This study was designed to assess treatment outcomes and determine predictors of poor treatment outcomes among patients with TB in war affected region of Khyber Pakhtunkhwa, Pakistan. We found that the overall TB treatment success (i.e. having an outcome of cured or treatment completed) at the end of the treatment was 86.2%, which is similar to other resource-constrained countries such as India (81–83%) [23–25], Ethiopia (85.6%) [26], Nigeria (83.1%) [27], Uzbekistan (83%) [28], Thailand (78.5–87.5%) [29], and higher than war affected countries such as Somalia (81.8%) [30], Iran (83.1%) [31] and Afghanistan (77.7%) [32]. However, the treatment success rate in our study was lower than the treatment success rate reported in the previous studies in Pakistan [13, 33]. It is also lower than the global End TB Strategy targets. Demographic and clinical factors such as type of TB, year of treatment commencement, and age, were significantly associated with treatment outcomes of patients with TB.

Our study showed that patients with age less than 60 years were nearly three times more likely to get successful treatment outcome as compared to patients with age greater than 61 years. These results are consistent with previous studies conducted in Ethiopia [34]. This may be due to the fact that older age patients are at a higher risk of death due to ageing. Another reason for low treatment outcome in older age patients could be because older age patients might be at higher risk of having chronic comorbidities such as cardiovascular diseases, hypertensions, and cancers. Low socio-economic status, poor adherence to treatment, and difficulty of traveling and arriving early at health facilities for DOTS could be also other reasons for low treatment outcomes in older age patients. These findings highlight the importance of providing close follow up for older age patients to increase their successful treatment outcome.

The other important finding is that smear positive pulmonary TB patients were more likely to have a successful treatment outcome than other types of TB patients. This result is consistent with previous studies conducted in Ethiopia [34]. This could be explained by the fact that smear positive pulmonary TB patients might be diagnosed easily and started the treatment promptly and may have close follow by health professionals. All TB diagnosed patients were treated at DOTS clinics using regimens recommended by WHO. Two types of treatment category were set CAT-I and CAT-II. Results of the current study show that patients with treatment CAT-I was more likely to have successful treatment outcomes than patients with treatment CAT-II.

Our study also showed that patients who started treatment before 2014 were more likely to have successful treatment outcomes than patients who started treatment in 2014. The finding indicated that successful treatment outcomes were decreased overtime from (87.0%) in 2011 and (94.9%) in 2012 to (73.9%) in 2014. This may be related to the desaturation of health care systems and TB treatment services of country because of the war. It could be also due to increased number of multidrug-resistant tuberculosis (MDR-TB) which is defined as TB that is resistant to the two most powerful first-line TB drugs (i.e. isoniazid and rifampicin). We also found that the overall prevalence of smear positive PTB was high among reported cases, which concurs with previous studies showing that majority of patients taking TB treatment were smear positive PTB [33]. The smear positive PTB patients are dangerous and can easily spread the infection in the community. Early diagnosis and treatment of such cases are very important and necessary to reduce the progression of TB. The overall case fatality rate was (2.72%) which is high from previous published studies [35]. The high rate of mortality among TB patients could be attributed to less access to hospitals, at door step availability of least health diagnostic facilities, no early diagnosis and treatment of the disease, poverty, and poor nutritional status in our society.

This study has several limitations. Firstly, since the study was based on secondary data, some important clinical variables such as HIV, diabetes mellitus, and other co-morbidities as well as behavioral factors such as alcohol drinking and smoking were not available in the registers and therefore were not included in our study. Secondly, in this study those patients who had documented evidence of completion were counted as having a successful treatment outcome, whereas they may have undetected failure of therapy. This may lead to overestimation of the treatment outcome rate in our study. Third, as the study used data reported between 2011 and 2014, we have not assessed recent treatment outcomes, and a longer follow-up period will be required to assess longer-term trends in treatment outcomes, which is out of the scope of the current study.

## Conclusion

The overall treatment success rate is satisfactory but still need to be improved to achieve the international targeted End TB Strategy milestones. Type of TB, age, treatment category, and year of enrollment were significantly associated with successful treatment outcomes. Successful treatment outcomes were decreased over time which is an alarming signal for MDR-TB.

## Data Availability

The datasets used, generated and/or analyzed in this study are available free of cost from the principal author/corresponding author on reasonable request.

## Abbreviations

TB: Tuberculosis
DHQ: District Head Quarter
AOR: Adjusted Odds Ratio
CI: Confidence Interval
HIV: Human Immunodeficiency Virus
AIDS: Acquired Immunodeficiency Syndrome
DOTS: Directly Observed Treatment Short-Course
WHO: World Health Organization
AFB: Acid Fast Bacilli
PTB: Pulmonary Tuberculosis
EPTB: Extra-pulmonary Tuberculosis
SPSS: Statistical Package for Social Sciences
CAT-I: Category-I
CAT- II: Category-II
COR: Crude Odds Ratio
MDR-TB: Multidrug-resistant Tuberculosis
